# Effectiveness of Omicron XBB.1.5 vaccine against infection with SARS-CoV-2 Omicron XBB and JN.1 variants, prospective cohort study, the Netherlands, October 2023 to January 2024

**DOI:** 10.2807/1560-7917.ES.2024.29.10.2400109

**Published:** 2024-03-07

**Authors:** Anne J Huiberts, Christina E Hoeve, Brechje de Gier, Jeroen Cremer, Bas van der Veer, Hester E de Melker, Janneke HHM van de Wijgert, Susan van den Hof, Dirk Eggink, Mirjam J Knol

**Affiliations:** 1 Centre for Infectious Disease Control, National Institute for Public Health and Environment (RIVM), Bilthoven, the Netherlands; 2 Julius Center for Health Sciences and Primary Care, University Medical Center Utrecht (UMCU), Utrecht, the Netherlands; * These authors contributed equally to this article and share last authorship

**Keywords:** COVID-19, vaccine effectiveness, SARS-CoV-2

## Abstract

We estimated vaccine effectiveness (VE) of SARS-CoV-2 Omicron XBB.1.5 vaccination against self-reported infection between 9 October 2023 and 9 January 2024 in 23,895 XBB.1.5 vaccine-eligible adults who had previously received at least one booster. VE was 41% (95% CI: 23–55) in 18–59-year-olds and 50% (95% CI: 44–56) in 60–85-year-olds. Sequencing data suggest lower protection against the BA.2.86 (including JN.1) variant from recent prior infection (OR = 2.8; 95% CI:1.2–6.5) and, not statistically significant, from XBB.1.5 vaccination (OR = 1.5; 95% CI:0.8–2.6).

A monovalent mRNA vaccine targeting the SARS-CoV-2 Omicron XBB.1.5 subvariant (Comirnaty; BioNTech-Pfizer, Germany/United States (US)) was used in the 2023 Dutch COVID-19 vaccination campaign that started on 2 October 2023. Individuals aged ≥ 60 years, medical risk groups, pregnant women and healthcare workers were eligible for vaccination. Since September 2023, a new Omicron BA.2.86 sub-variant named JN.1 has emerged and quickly become dominant [1,2]. The JN.1 variant is genetically divergent from the previously circulating XBB variants, indicating potential for immune escape [3].

We estimated vaccine effectiveness (VE) of XBB.1.5 vaccination against self-reported SARS-CoV-2 infection between 9 October 2023 and 9 January 2024 among adults aged 18–85 years who had previously received primary vaccination and at least one booster vaccination before 2 October 2023 and were eligible for XBB.1.5 vaccination. To assess potential lower protection against JN.1, we analysed whether there was an association between XBB.1.5 vaccination or prior infection and the Omicron variant causing the infection (XBB vs BA.2.86, including JN.1). We determined the variant by sequencing viral genetic material present in positive antigen self-tests [4,5].

## Study population

We used data from 23,895 participants of the VAccine Study COvid-19 (VASCO), an ongoing prospective cohort study among community-dwelling Dutch adults aged 18–85 years that started in May 2021 [6,7]. Participants are followed with 3-monthly follow-up questionnaires and 6-monthly self-collected fingerprick blood samples for serological testing. Participants can report COVID-19 vaccinations and positive SARS-CoV-2 tests (PCR or antigen (self-)test) any time. Self-tests are provided to participants to facilitate testing in case of COVID-19-like symptoms. For the current analysis, follow-up time started on 9 October 2023 and ended on 9 January 2024, on the date of first positive SARS-CoV-2 test or on the date of last completed follow-up questionnaire, whichever occurred first. The 7 person-days after XBB.1.5 vaccination were excluded. If participants reported a vaccination or infection in the 3 months before the XBB.1.5 vaccination campaign, follow-up started 3 months after this vaccination or infection as people were eligible for XBB.1.5 vaccination earliest 3 months after prior infection or vaccination.

Of 4,861 participants aged 18–59 years who were eligible to receive the XBB.1.5 vaccine because they were a healthcare worker or belonged to a medical risk group, 1,167 (24%) received the XBB.1.5 vaccination during the follow-up period (Table 1). Of 19,034 participants aged 60–85 years, eligible for the XBB.1.5 vaccine because of age, 11,330 participants (60%) received the XBB.1.5 vaccination.

**Table 1:** Characteristics of included VASCO participants vaccinated with COVID-19 primary series, at least one booster dose and eligible for XBB.1.5 vaccination, the Netherlands, October 2023–January 2024 (n = 23,895)

	18–59 years						60–85 years					
	Total		Without XBB.1.5 vaccination		With XBB.1.5 vaccination		Total		Without XBB.1.5 vaccination		With XBB.1.5 vaccination	
	n	%	n	%	n	%	n	%	n	%	n	%
All participants^a^	4,861	100	3,694	100	1,167	100	19,034	100	7,704	100	11,330	100
Median age in years (IQR)	51 (42–56)		51 (41–55)		52 (44–56)		66 (64–70)		65 (63–69)		67 (65–71)	
Sex												
Female	4,034	83.0	3,060	82.8	974	83.5	10,623	55.8	4,524	58.7	6,099	53.8
Male	823	16.9	632	17.1	191	16.4	8,411	44.2	3,180	41.3	5,231	46.2
Other	4	0.1	2	0.1	2	0.2	0	0	0	0	0	0
Medical risk condition^b^												
Yes	2,688	55.3	1,992	53.9	696	59.6	8,179	43.0	3,075	39.9	5,104	45.0
Cardiovascular disease	889	18.3	659	17.8	230	19.7	4,920	25.8	1,805	23.4	3,115	27.5
Lung disease or asthma	869	17.9	588	15.9	281	24.1	1,470	7.7	520	6.7	950	8.4
Diabetes mellitus	246	5.1	163	4.4	83	7.1	1,229	6.5	450	5.8	779	6.9
Immunodeficiency	205	4.2	146	4.0	59	5.1	307	1.6	124	1.6	183	1.6
Healthcare worker												
Yes	2,832	58.3	2,156	58.4	676	57.9	2,106	11.1	985	12.8	1,121	9.9
Education level^c^												
High	2,995	61.6	2,222	60.2	773	66.2	10,193	53.6	3,779	49.1	6,414	56.6
Intermediate	1,583	32.6	1,237	33.5	346	29.6	5,125	26.9	2,254	29.3	2,871	25.3
Low	262	5.4	215	5.8	47	4.0	3,577	18.8	1,615	21.0	1,962	17.3
Other	21	0.4	20	0.5	1	0.1	139	0.7	56	0.7	83	0.7
Bivalent booster in the autumn 2022 vaccination campaign (started on 19 September 2022)												
Yes	3,121	64.2	2,062	55.8	1,059	90.7	15,799	83.0	5,222	67.8	10,577	93.4
No	1,740	35.8	1,632	44.2	108	9.3	3235	17.0	2,482	32.2	753	6.6
Median time since last booster vaccination at start of study follow-up												
Weeks (IQR)	51.7 (49–90)		52.6 (49–91)		50.7 (49–52)		50.7 (49–52)		50.9 (49–66)		50.6 (49–52)	
SARS-CoV-2 infection history^d^												
No prior infection	399	8.2	268	7.3	131	11.2	3,130	16.4	1,184	15.4	1,946	17.2
Infection ≥ 1 year ago	2,973	61.2	2,302	62.3	671	57.5	10,558	55.5	4,341	56.3	6,217	54.9
Infection < 1 year ago	1,489	30.6	1,124	30.4	365	31.3	5,346	28.1	2,179	28.3	3,167	28.0
Test intention ((almost) always testing when having symptoms)												
Yes	3,614	74.3	2,668	72.2	946	81.1	16,604	87.2	6,551	85.0	10,053	88.7
Most recent serological data^e^												
< 1 year before start of follow-up	4,106	84.5	3,111	84.2	995	85.3	16,465	86.5	6,666	86.5	9,799	86.5
> 1 year before start of follow-up	700	14.4	541	14.6	159	13.6	2,283	12.0	908	11.8	1,375	12.1
No serological data available	55	1.1	42	1.1	13	1.1	286	1.5	130	1.7	156	1.4

IQR: interquartile range; N: nucleoprotein; SARS-CoV-2: severe acute respiratory syndrome coronavirus 2; VASCO: Vaccine Study Covid-19.

^a^ Participants who had previously received primary vaccination series and at least one booster vaccination and were eligible for XBB.1.5 vaccination (aged ≥ 60 years, medical risk groups and healthcare workers). Participants who received a third dose as part of their primary series before the start of the first general public booster campaign (18 November 2021) were excluded.

^b^ Medical risk condition: one or more of the following conditions: diabetes mellitus, lung disease or asthma, asplenia, cardiovascular disease, immunodeficiency, cancer, liver disease, neurological disease, renal disease, organ or bone marrow transplantation.

^c^ Educational level was classified as low (no education or primary education), intermediate (secondary school or vocational training) or high (bachelor’s degree).

^d^ Most recent prior SARS-CoV-2 infection based on self-reported positive SARS-CoV-2 tests or anti-N antibodies. When based on anti-N antibodies (no corresponding infection reported), infection date was imputed as mid-date between two blood samples, where the first was negative and the second was positive for N antibodies, or where the second had at least a fourfold increase in N antibody concentration compared with the first. When the first serum sample of a participant was positive for N antibodies but no prior infection was reported, an infection date was imputed as the mid‐date between the baseline questionnaire and sample receipt.

^e^ Most recent fingerprick blood sample for which anti-N antibodies could be determined. Participants are requested to take a fingerprick blood sample every 6 months during follow-up.

**Figure 1:**
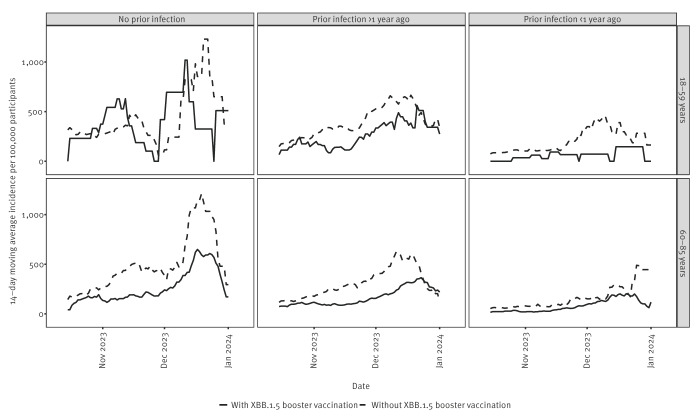
14-day moving average of the number of reported infections per 100,000 participants, by infection history and age group, the Netherlands, 9 October 2023–9 January 2024 (n = 23,895)

During follow-up, 1,951 SARS-CoV-2 infections were reported. Reported incidence increased during the study period (Figure 1), consistent with national syndromic and wastewater surveillance data [8,9]. Those without prior infection, defined as not having any self-reported positive SARS-CoV-2 test and not having any positive anti-nucleoprotein serology (8% of 18–59-year-olds; 16% of 60–85-year-olds), had the highest incidence (note that the 18–59-year-old group included a low number of participants and infections). The number of participants and infections are appended in Supplementary Figure S1.

## Vaccine effectiveness

We estimated VE using Cox proportional hazard models with calendar time as time scale, XBB.1.5-vaccination as time-varying exposure and adjustment for age group, sex, education level, medical risk condition and infection history. Analyses were performed using R version 4.3.2, packages Epi, survival and stats.

**Figure 2:**
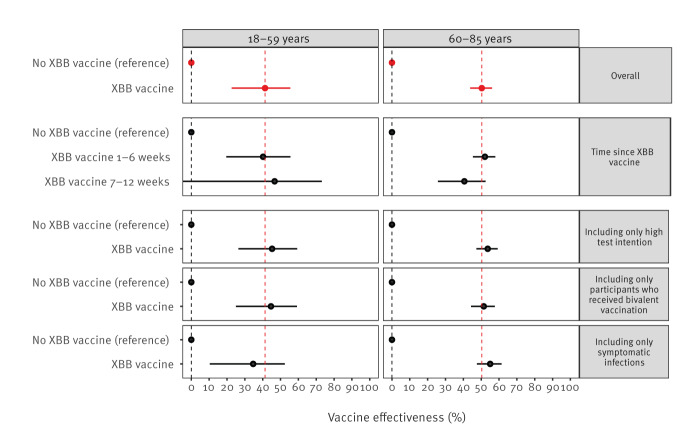
Vaccine effectiveness^a^ of XBB.1.5 vaccine against SARS-CoV-2 Omicron infection among participants vaccinated with primary series and at least one booster and eligible for XBB.1.5 vaccination, stratified by age group, the Netherlands, 9 October 2023–9 January 2024 (n =23,895)

The VE was 41% (95% CI: 23–55) among 18–59-year-olds and 50% (95% CI: 44–56) among 60–85-year-olds (Figure 2). We provide incidence rates in Supplementary Table S1. The VE estimates were slightly higher up to 6 weeks after vaccination compared with 7–12 weeks after vaccination among 60–85-year-olds (52% vs 41%), but CIs were wide. A sensitivity analysis among participants who, during the study period, reported to (almost) always test in case of symptoms showed a slightly higher VE (Figure 2). For additional detail on VE estimates of sensitivity analyses we refer to Supplementary Table S2. When only known symptomatic infections (68% of the infections; 21% asymptomatic, 11% unknown) were included, VE was 35% (95% CI: 10–52) among 18–59-year-olds and 55% (95% CI: 48–61) among 60–85-year-olds.

SARS-CoV-2: severe acute respiratory syndrome coronavirus 2.

^a^ Adjusted for age group (18–39, 40–59, 60–69, 70–85 years), sex, education level, presence of a medical risk condition and infection history.

Whiskers indicate 95% confidence intervals.

Irrespective of infection history, XBB.1.5 vaccination provided additional protection in 18–59-year-olds and 60–85-year-olds. Among 60–85-year-olds, protection against infection was highest after a prior infection < 1 year ago either with (85%) or without (73%) XBB.1.5 vaccination; these estimates are appended in Supplementary Table S2. This same trend was seen in 18–59-year-olds (87% and 61%, respectively).

## Emerging subvariant

From June 2023, VASCO participants were asked to submit positive SARS-CoV-2 self-tests for sequencing. Residual viral genetic material was extracted from the test strips of the self-tests showing a clear positive result (as described in [5]), PCR-amplified and sequenced [4].

Of 3,525 reported infections between 9 October 2023 and 9 January 2024 (also including infections notified after completion of the last questionnaire and therefore excluded from the VE analysis), we sequenced material extracted and amplified from 764 self-tests with a strong positive band. Of those, 530 resulted in (near) whole genome sequences (> 90% coverage) and an additional 63 resulted in enough coverage (70–90%) to allow variant typing using Pangolin; a flowchart of total number of infections and sequenced self-tests is appended in Supplementary Figure S2. Identified variants were Omicron XBB (n = 173; 29%), Omicron BA.2.86 (n = 407; 69%), of which 342 (84%) were sub-variant JN.1, and other Omicron variants (n = 13; 2%). Infections caused by BA.2.86 increased from 6% in the first week of the study period to 100% in the last week; we provide the detailed results in Supplementary Figure S3. Observed prevalence of variants corresponded well with national SARS-CoV-2 genomic surveillance [10].

Among infected participants with sequencing data, we performed logistic regression to estimate the association between XBB.1.5 vaccination or prior infection and the Omicron variant causing the infection (XBB or BA.2.86), adjusting for testing week, age group, sex, education level and medical risk condition. For the characteristics of these participants we refer to the appended Supplementary Table S3).

Participants who had received the XBB.1.5 vaccine had slightly (though non-significantly) higher odds of their infection being caused by BA.2.86 and JN.1 sub-variants rather than XBB variants (OR = 1.5; 95% CI: 0.8–2.6); Table 2), suggesting that the VE against BA.2.86 is only slightly lower than the VE against XBB. Participants with a prior infection > 1 year ago had minor and non-significantly higher odds of their infection being caused by BA.2.86 rather than XBB (OR = 1.5; 95% CI: 0.8–2.8), compared with participants without prior infection. Participants with a prior infection < 1 year ago had significantly higher odds of their infection being caused by BA.2.86 rather than XBB (OR = 2.8; 95% CI: 1.2–6.5). A subgroup analysis including only JN.1 and descendants resulted in comparable estimates for vaccination (OR = 1.3; 95% CI: 0.7–2.5), prior infection > 1 year ago (OR = 1.4; 95% CI: 0.7–2.8) and prior infection < 1 year ago (OR = 2.8; 95% CI: 1.1–7.2).

**Table 2:** Association between XBB.1.5 vaccination status and SARS-CoV-2 BA.2.86 vs XBB infection, the Netherlands, 9 October 2023–9 January 2024 (n = 580)

	XBB infection (n = 173)		BA.2.86 infection (n = 407)		Adjusted^a^ OR (95% CI)
	n	%	n	%	
XBB.1.5 vaccination status					
Without XBB.1.5 vaccination	138	79.8	196	48.2	Reference
With XBB.1.5 vaccination	35	20.2	211	51.8	1.5 (0.8–2.6)
Infection history					
No prior infection	43	24.9	67	16.5	Reference
Infection > 1 year ago	111	64.2	257	63.1	1.5 (0.8–2.8)
Infection < 1 year ago	19	11.0	83	20.4	2.8 (1.2–6.5)

OR: odds ratio; SARS-CoV-2: severe acute respiratory syndrome coronavirus 2.

^a^ Adjusted for testing week, age group (18–39, 40–59, 60–69, 70–85 years), sex, education level and presence of a medical risk condition.

## Discussion

Other studies have shown benefit of the XBB.1.5 vaccination campaign in reducing COVID-19 hospitalisations [18,19]. Here we report considerable protection against SARS-CoV-2 infection in the first 3 months after XBB.1.5 vaccination. The VE of XBB.1.5 vaccination against self-reported SARS-CoV-2 infection among XBB.1.5 vaccine-eligible participants was 41% for 18–59-year-olds and 50% for 60–85-year-olds, with similar estimates against symptomatic infection. Furthermore, irrespective of vaccination status, protection conferred by a recent prior infection against a new infection was high. Protection from XBB.1.5 vaccination and recent prior infection seemed slightly lower against BA.2.86 and sub-variant JN.1 compared with XBB infection (OR = 1.5 (non-significant) and OR = 2.8, respectively).

A recent study from the US reported comparable VE estimates against symptomatic infection of 57% in 18–49-year-olds and 46% in those aged ≥ 50 years [11]. Their VE estimates were considerably higher than their estimates during the 2022/23 winter season of the bivalent booster vaccination (46%, 38% and 36% in individuals aged 18–49 years, 50–64 and ≥ 65 years, respectively) [12]. Also our estimates described here were considerably higher than our estimates of the bivalent booster in 2022: 31% in 18–59-year-olds and 14% in 60–85-year-olds [13]. This suggests that the 2023 XBB.1.5 vaccine better matches the virus that was circulating in the 2023/24 winter season than the 2022 bivalent vaccine did in the 2022/23 winter season, when Omicron BQ.1 and BA.2.75 were circulating in the Netherlands [10]. Moreover, while the XBB.1.5 is a monovalent vaccine targeting the Omicron XBB.1.5 subvariant, the bivalent booster included the original Wuhan strain of SARS-CoV-2 and the Omicron BA.1 subvariant. This could have skewed response towards the original strain, diminishing the effectiveness of the response to the Omicron BA.1 subvariant in the vaccine. Possible alternative explanations for the higher VE compared with the previous year are the longer time period since prior vaccination campaigns and lower virus circulation in the summer of 2023. However, sensitivity analyses showed similar VEs among those who had received the bivalent vaccination, and after stratifying by infection history.

Preliminary immunological data suggest that BA.2.86 and sub-variant JN.1 (we took BA.2.86 and its descendants as one group to increase power for our analysis) show modest signs of immune evasion to XBB.1.5 vaccination but can still be neutralised by XBB.1.5 vaccine-induced antibodies [14,15] and prior XBB infection [16]. A recent study from Denmark reported that XBB.1.5-vaccinated cases had 1.6 times higher odds of their infection being caused by BA.2.86 than other variants compared with unvaccinated cases [17], consistent with our findings. Also, VE estimates of XBB.1.5 vaccination reported in the US were slightly lower against infections caused by JN.1 (49%) compared with XBB-related lineages (60%), although the difference was non-significant [11].

We provided participants with self-tests free of charge, thereby reducing bias associated with access to testing. We did rely on participant adherence to study instructions related to testing and reporting of infections, which could have influenced our estimates. However, restricting the analysis to participants with high test intention only slightly increased our estimates. Serological data enabled us to detect and adjust for prior untested (asymptomatic) infections, but serology data were not available for all study participants (84–87% of participants in the past year). Furthermore, imputation of the infection date when an infection was detected by serology only may have resulted in misclassification of time since prior infection. Self-reported vaccination data was confirmed by linkage to vaccination registry data, limiting exposure misclassification.

## Conclusion

We showed that XBB.1.5 vaccination provided considerable protection against SARS-CoV-2 infection in the first 3 months after vaccination. Recent prior infection also protects against new infection, but it should be kept in mind that experiencing a SARS-CoV-2 infection carries a risk of severe disease in vulnerable groups, and of post-COVID condition. We found indications of lower protection against the emerging BA.2.86 (JN.1) variant, suggesting possible immune escape from XBB.1.5 vaccination and prior infection, which could have contributed to the rapid increase of this variant worldwide. 

## Supplementary Data

515000 Supplement
