# Supplementary material for: Effectiveness of Omicron XBB.1.5 vaccine against infection with SARS-CoV-2 Omicron XBB and JN.1 variants, prospective cohort study, the Netherlands, October 2023 to January 2024
Source: Euro Surveill. 2024 Mar 7;29(10):2400109. doi: 10.2807/1560-7917.ES.2024.29.10.2400109 (PMC10986669; doi:10.2807/1560-7917.ES.2024.29.10.2400109)
Supplement: Supplement [file 24-00109_KNOL_Supplement.pdf]

Supplementary file - Effectiveness of Omicron XBB.1.5 vaccine against infection with SARS-CoV-2 Omicron XBB and JN.1 variants, prospective cohort study, the Netherlands, October 2023 to January 2024

This supplementary material is hosted by Eurosurveillance as supporting information alongside the article [Effectiveness of Omicron XBB.1.5 vaccine against infection with SARS-CoV-2 Omicron XBB and JN.1 variants, prospective cohort study, the Netherlands, October 2023 to January 2024], on behalf of the authors, who remain responsible for the accuracy and appropriateness of the content. The same standards for ethics, copyright, attributions and permissions as for the article apply. Supplements are not edited by Eurosurveillance and the journal is not responsible for the maintenance of any links or email addresses provided therein.

Figure S1. Number of participants and reported SARS-CoV-2 infections in the period 9 October 2023 to 9 January 2024, by vaccination status, infection history and age group

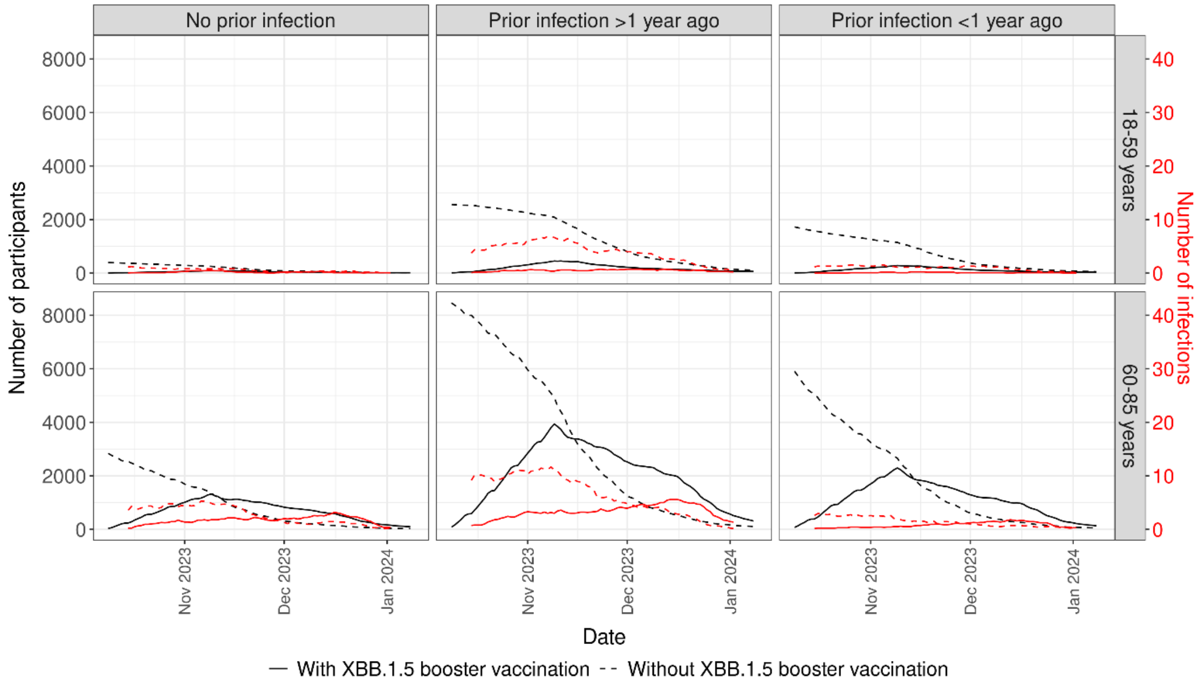

Table S1. Incidence rate and vaccine effectiveness of XBB.1.5 vaccination in the period 9 October 2023 to 9 January 2024, stratified by age group

	Person-weeks	Number of infections	Rate (per 1,000 weeks)	Adjusted[a] vaccine effectiveness (%) (95% CI)
**18-59 years**				
**Overall[b]**				
Without XBB vaccination	28667	475	16.6	Reference
With XBB vaccination	4705	59	12.5	41.3 (22.6-55.5)
**By time since XBB vaccination**				
Without XBB vaccination	28667	475	16.6	Reference
With XBB vaccination (1-6 weeks)	4145	50	12.1	40.2 (19.6-55.5)
With XBB vaccination (7-12 weeks)	552	9	16.3	46.7 (-5.7-73.1)
**Participants who (almost) always test in case of symptoms**				
Without XBB vaccination	20223	397	19.6	Reference
With XBB vaccination	3704	52	14.0	45.3 (26.4-59.3)
**Participants who received a bivalent booster in autumn 2022 COVID-19 vaccination campaign (started on 19 September 2022)**				
Without XBB vaccination	16492	284	17.2	Reference
With XBB vaccination	4274	53	12.4	44.6 (25.0-59.1)
**Symptomatic infections only**				
Without XBB vaccination	28667	330	11.5	Reference
With XBB vaccination	4705	46	9.8	34.7 (10.4-52.4)
**60-85 years**				
**Overall**				
Without XBB vaccination	78581	939	11.9	Reference
With XBB vaccination	49512	478	9.7	50.3 (43.8-56.1)
**By time since XBB vaccination**				
Without XBB vaccination	78581	939	11.9	Reference
With XBB vaccination (1-6 weeks)	42819	356	8.3	52.1 (45.4-57.9)
With XBB vaccination (7-12 weeks)	6666	122	18.3	40.6 (25.7-52.4)
**Participants who (almost) always test in case of symptoms**				
Without XBB vaccination	66839	878	13.1	Reference
With XBB vaccination	43742	448	10.2	53.7 (47.3-59.2)
**Participants who received a bivalent booster in autumn 2022 COVID-19 vaccination campaign (started on 19 September 2022)**				
Without XBB vaccination	58948	706	12.0	Reference
With XBB vaccination	46591	458	9.8	51.4 (44.3-57.6)
**Symptomatic infections only**				
Without XBB vaccination	78581	648	8.2	Reference
With XBB vaccination	49512	298	6.0	55.0 (47.6-61.4)

[a] Adjusted for age group (18-39, 40-59, 60-69, 70-85), sex, education level, medical risk condition and infection history.

[b] Interaction between XBB.1.5 vaccination and infection history was not significant (p=0.17 and p=0.77 for 18-59 and 60-85 year-olds, respectively); therefore results were not stratified by infection history

Table S2. Protection[a] by XBB.1.5 vaccination and prior infection in the period 9 October 2023 to 9 January 2024, stratified by age group

	**No prior infection**	**Prior infection >1 year ago**	**Prior infection <1 year ago**
**18-59 years**			
Without XBB.1.5 vaccination	Reference	14.0 (-16.5-36.6)	61.3 (44.8-72.9)
With XBB.1.5 vaccination	11.7 (-60.9-51.6)	49.7 (22.8-67.2)	86.7 (68.9-94.3)
**60-85 years**			
Without XBB.1.5 vaccination	Reference	32.9 (21.8-42.3)	72.6 (66-77.9)
With XBB.1.5 vaccination	48.8 (36.4-58.8)	67.7 (61.2-73.1)	85.3 (80.6-88.9)

[a] Adjusted for age group (18-39, 40-59, 60-69, 70-85 years), sex, education level and medical risk condition

Figure S2. Flowchart of total number of infections and sequenced self-tests in the period 9 October 2023 to 9 January 2024

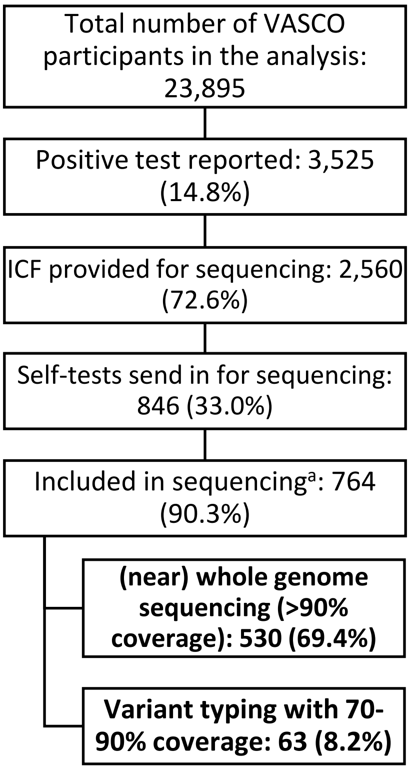

[a] Weak-positive self-tests were not included in sequencing

Figure S3. Variants of infection based on sequencing of self-tests per week from 9 October 2023 to 9 January 2024

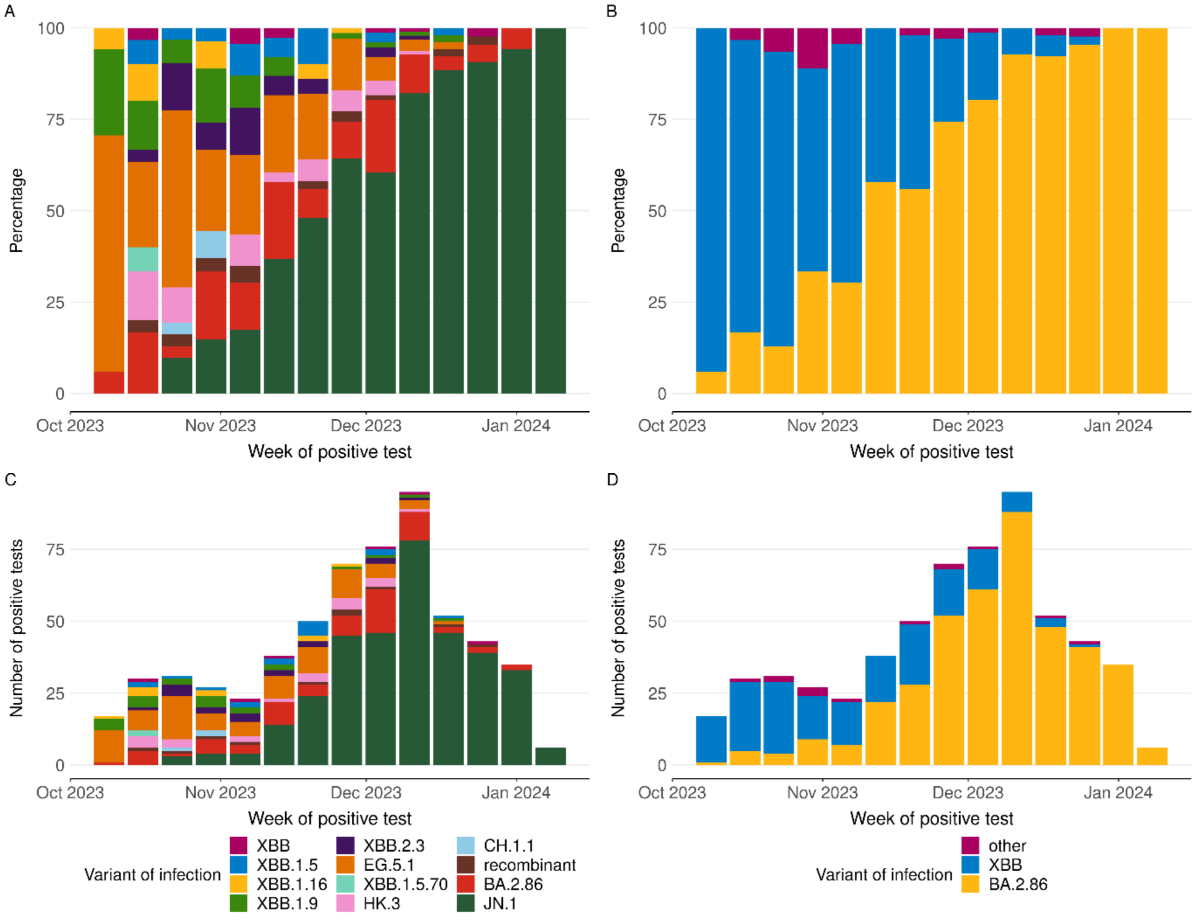

A) and C) SARS-COV-2 subvariants of infection observed during the study period; B) and D) general classification of subvariants of infection observed during the study period, with all XBB subvariants (XBB, XBB.1.5, XBB.1.16, XBB.1.9, XBB.2.3, EG.5.1, XBB.1.5.70, HK.3) and all BA.2.86 derivatives (BA.2.86, JN.1, JN.2, JN.3, JN.4, JN.5, JN.6, JN.9, JN.10 and subvariants) grouped together for the XBB vs BA.2.86 analysis.

Table S3. Demographics of participants by variant of infection based on sequencing of self-tests in the period 9 October 2023 to 9 January 2024

	**Total**		**BA.2.86 infection**		**XBB infection**		**P-value**
	n	%	n	%	n	%	
**All participants**	580	100	407	100	173	100	
**Age (years)**							
18-59 years	195	33.6	127	31.2	68	39.3	0.073
60-85 years	385	66.4	280	68.8	105	60.7	
**Sex**							
Female	413	71.2	294	72.2	119	68.8	0.460
Male	167	28.8	113	27.8	54	31.2	
**Medical risk condition, yes**	237	40.9	169	41.5	68	39.3	0.686
**Education level**							
High	348	60.0	240	59.0	108	62.4	0.394
Intermediate	160	27.6	112	27.5	48	27.7	
Low	68	11.7	53	13.0	15	8.7	
Other	4	0.7	2	0.5	2	1.2	
**XBB.1.5 vaccination**							
Yes	246	42.4	211	51.8	35	20.2	<0.001
No	334	57.6	196	48.2	138	79.8	
**Infection history**							
No prior infection	102	19.0	67	16.5	43	24.9	0.005
Infection > 1 year ago	368	63.4	257	63.1	11	64.2	
Infection < 1 year ago	110	17.5	83	20.4	19	11.0	
